# Fascioliasis: An Ongoing Zoonotic Trematode Infection

**DOI:** 10.1155/2015/786195

**Published:** 2015-08-31

**Authors:** Mramba Nyindo, Abdul-Hamid Lukambagire

**Affiliations:** Department of Medical Parasitology and Entomology, Kilimanjaro Christian Medical University College, P.O. Box 2240, Moshi, Tanzania

## Abstract

Zoonotic trematode infections are an area of the neglected tropical diseases that have become of major interest to global and public health due to their associated morbidity. Human fascioliasis is a trematode zoonosis of interest in public health. It affects approximately 50 million people worldwide and over 180 million are at risk of infection in both developed and underdeveloped countries. The one health paradigm is an area that seeks to address the problem of zoonotic infections through a comprehensive and sustainable approach. This review attempts to address the major challenges in managing human and animal fascioliasis with valuable insights gained from the one health paradigm to global health and multidisciplinary integration.

## 1. Introduction

Fascioliasis is a disease of ruminants caused by two major parasitic trematodes,* Fasciola hepatica* and* F. gigantica*. Over the past two decades human fascioliasis has gained notice as a disease of primary importance. Human fascioliasis is currently classified as a plant/food-borne trematode infection, commonly acquired by eating metacercaria encysted on leaves that are eaten as vegetables [1]. There is a high prevalence of fascioliasis among herding communities in low income countries because of their constant close association with livestock that they keep.

Fascioliasis was first recorded as early as 2000 BC. Animal fascioliasis causes significant disease among sheep and cattle, causing severe physical wasting. It contributes to losses of over $2 billion dollars per annum in the livestock industry in North and South America. Human fascioliasis also causes significant illness and morbidity, mainly among low income, farming communities. To date, no human deaths have been directly associated with fascioliasis. This fact accords the disease a low priority and contributes to its neglect as a significant cause of public health concern. Human fascioliasis is currently ranked under the food/plant trematode zoonoses as a neglected tropical disease (NTD) [2].

## 2. Materials and Methods

We conducted a literature search on human and animal fascioliasis in the past 15 years. We searched PubMed/MEDLINE resources, HINARI/PubMed, local, regional, and institutional e-libraries such as NIH, CDC, WHO, and IHI (Ifakara Health Institute, Tanzania) e-lib, East African Medical Journal, and African journals of public health as well as Vectors and Zoonoses for the following major keywords: “fascioliasis,” “human fascioliasis,” “Zoonoses,” “fasciola liver fluke,” “fasciola hepatica,” “fasciola gigantica,” “fascioliasis, F. hepatica,” “fascioliasis F. gigantica,” “human fascioliasis epidemiology,” “human fascioliasis distribution,” “trends in fascioliasis research,” and “fascioliasis outbreaks.” We searched published literature from 2000 to 2015. Also we referenced recognized full textbook articles as well as summaries and abstracts containing discernible content. We selected references that fitted to being included in the review. We excluded non-English translated articles and papers/publications that only focused on animal disease without a zoonotic aspect. This search allowed us to include 92 most relevant references out of 300.

## 3. Current Status

Fascioliasis is primarily a disease of ruminants, although over the past two decades human fascioliasis has gained significance as an important disease in humans [1]. Human fascioliasis is commonly characterized by a hypoendemic pattern, with low and stable levels of prevalence among a defined population, and generally shows a focal endemic distribution. However, to date, there have been reports from every continent except the Antarctica, thereby showing a wide cosmopolitan distribution [2].

Among the zoonotic parasitic diseases, human fascioliasis is currently classified as a plant/food-borne trematode infection, with higher prevalence seen among farming communities in low income countries [3, 4]. The plant/food-borne trematodes comprise* Fasciola hepatica, F. gigantica,* and* Fasciolopsis buski* (family Fasciolidae),* Gastrodiscoides hominis* (family Gastrodiscidae),* Watsonius watsoni,* and* Fischoederius elongatus* (Family Paramphistomidae).* Fasciola hepatica* and* F. gigantica* infect the liver [5].

The fasciolids and the gastrodiscid cause important zoonoses distributed throughout many countries, while* W. watsoni* and* F. elongatus* have been only accidentally transmitted to humans. Present climate and global changes appear to increasingly affect the distribution of snail-borne helminthiases [6]. This provides a good example of emerging/reemerging parasitic diseases in many countries as a consequence of various phenomena related to climatic and environmental changes as well as human socioeconomic activities [5, 7].

Despite the recent developments in diagnostic and surveillance techniques, some countries are still completely lacking in data on human fascioliasis. This may be because the disease is not endemic but is more likely due to underreporting/diagnosis especially in the resource limited settings. The underestimated global burden of the disease to date is approximated to be between 35 and 72 million people, with an additional 180 million at risk of infection [8–10]. Abundant data supporting animal* Fasciola* infections are available in many tropical developing countries and regions, with the corresponding presence of snail species responsible for transmission. Therefore, the possibility of transmission of animal fascioliasis to humans is high where close proximity of humans with domestic animals is common [3, 6].

Although suitable environment and interactions for transmission have been established in many potentially endemic areas, reliable diagnostic and surveillance methods to establish presence of human fascioliasis are usually lacking [2, 11, 12]. The apparent rarity of human fascioliasis infection in such areas underestimates the prevalence of the disease [9]. Because local physicians may not be fully informed about human fascioliasis, they may mistake fascioliasis for other diseases with similar clinical picture [3, 13].

### 3.1. Distribution of Human Fascioliasis

To date, human fascioliasis has been identified in many countries. The highest prevalence has been reported in Bolivia, Peru, Cuba, China, Spain, Nile Delta in Egypt, central areas of Vietnam, and Northern Iran [1, 14]. Bovine fascioliasis accounts for the majority of transmissions and is evenly spread around the world causing 29% of zoonoses [15]. Hyperendemicity of human fascioliasis has been noted in the Middle East and North African (MENA) region [16, 17] particularly in Egypt [10, 18], Ethiopia [19], Iran [14, 20], Iraq [21], Syria [16], and Saudi Arabia [2].

The highest prevalence has been reported in the South American highlands of Bolivia and Peru [9, 22]. High prevalence of human fascioliasis has been associated with recent climate changes, human settlement, and socioeconomic activities [4, 7, 23]. Also in South America, Argentina [3], Brazil [24], and Mexico [25] have recently reported epidemics of human fascioliasis. In South East Asia (SEA) and the Indian subcontinent, Cambodia [7], China [26–28], Vietnam [5], Singapore [29], Philippines, and India [30–32] have also recently reported rising case numbers of the disease that were previously unseen at country or regional health systems.

### 3.2. Human Fascioliasis in Europe and America

Europe has also had a long history of the disease in Italy [33, 34] and Spain [1, 11], Turkey [35–37], Britain [38, 39], France [6], and Greece [40]. Scattered cases have also been reported from North America [41] and Cuba [42, 43]. According to recent reviews [1, 44, 45], fascioliasis is probably the most widespread parasitic infection worldwide. With increased travel, open free trade, coupled with economic activities, and rural-urban human migration, fascioliasis is set to become a major disease of interest in public health, travel, and trade medicine [15, 45–48].

### 3.3. Life Cycle of Fasciola

Knowledge of the life cycle of a parasite may contribute to control strategies focusing on either the mammalian host or the vector. Infected mammals including not only cattle, sheep, buffaloes, donkeys, and pigs but also horses, goats, dromedaries, camels, llamas, and other herbivores pass ovulated eggs in stool into fresh-water sources [12, 19]. Since the* Fasciola* worm lives in the bile ducts of such animals, its unembryonated eggs reach the intestine with bile and are voided with feces. Fresh water is required for the development of intermediate stages of the* Fasciola* species in the snail. The ciliated miracidium hatches from the egg. It bores a snail in the genus* Lymnaea* and develops into a sporocyst. The next developmental stages are redia and cercaria [5, 9] which later vacate the snail. The cercaria can infect the definitive mammalian host, including humans passively when the host drinks infected water, or it can encyst on leaves and the mammalian host becomes infected when it eats leaves containing the metacercariae [16].

The ingested metacercariae excyst in the duodenum and migrate into the peritoneal cavity and finally reach the liver. They bore through the liver capsule and in about 12 weeks enter the bile ducts where they start to lay eggs. Infected persons develop hyperplasia of the bile ducts. Clinically, patients lose appetite and have nausea and diarrhea. Urticaria, acute epigastric pain, jaundice, eosinophilia, and hepatomegaly are common findings. In the chronic phase of the disease, hyperplasia of the gall bladder and biliary epithelium occurs and this leads to biliary tract obstruction. When live adult worms in an infected liver lodge in the throat region, they cause discomfort. After a period of about one to two months, a hypersensitivity reaction in the pharyngeal area develops. The term Halzoun syndrome describes the resulting suffocative immunological reaction at the pharyngeal area [6, 11, 16].

Factors noted to contribute to increased human transmission of fascioliasis include (i) high density of both human and animal populations living in close proximity, (ii) the presence of abattoirs and wet markets operating with rudimentary hygiene, limited cold chain for distribution, and low levels of meat inspection and biosafety measures, (iii) widespread consumption of raw/undercooked blood, meat, organ tissues, and offals and consumption of raw leaf vegetables, and (iv) the use untreated water sources for household use and/or use of untreated wastewater and sewage for agriculture [5, 6].

### 3.4. Snail Vectors and Distribution of Fascioliasis in Africa

The distribution of the* F. hepatica* and* F*.* gigantica* parasites is ubiquitous, mainly attributed to and associated with the equally global distribution of the viable, intermediate fresh-water snail hosts [3, 5]. Species distribution of the lymnaeid snails may be generalized as mainly temperate, at a higher altitude over 2500 m above sea level for* Lymnaea truncatula, L. rubiginosa,* and their associated parasite,* F. hepatica,* while* L. rupestris* and* L. natalensis* alongside their typical parasite* F. gigantica* have a more tropical/subtropical distribution at lower altitudes, below 2000 m [17, 49, 50].

In several countries in Africa and Asia it should be considered that* F. hepatica* and* F. gigantica* coexist, notably in areas of the Nile drainage, the great lakes mountain ranges, and the rift valley arms. In such environments, alternating altitudes and climatic conditions favour the respective snail vectors [4, 11, 50]. The differential specific diagnosis relating to eggs and specific antigens is of interest because of their different transmission, epidemiology, and control measures [51]. Mixed infections and hybridization have also been cited recently [52–54].

Surveys done in the hyperendemic Nile Delta valley in Egypt [4, 18] and river Tana basin in Ethiopia [19] found a high association between fascioliasis and schistosomiasis as well as myriad other intestinal parasites [19]. A gender distribution skewed in both intensity and prevalence towards girls in the age group of 9–11 years among a young key population was also noted. The coinfection and childhood distribution raise a further differential in the clinical presentation and etiology of parasitic illnesses on the continent especially in rural, animal rearing areas [4, 19].

**Figure 1:**
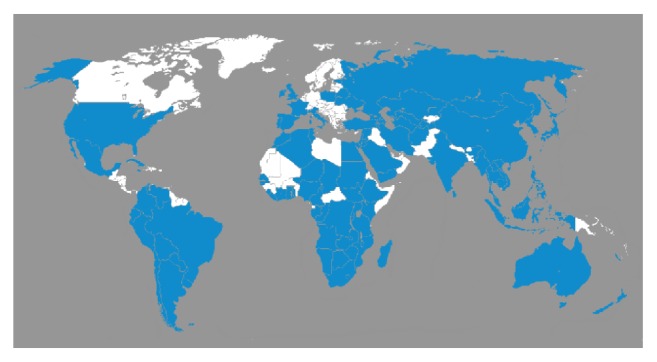
Global distribution of fascioliasis, 2015, countries reporting cases of fascioliasis shaded in blue (http://www.bvgh.org/Current-Programs/Neglected-Disease-Product-Pipelines/Global-Health-Primer/Diseases/cid/ViewDetails/ItemID/23.aspx). The figure implies that fascioliasis is distributed in over 90% of the world [84].

Other African countries reporting scattered cases of human fascioliasis include Cameroon [55], Chad [56], Senegal [57], South Africa [58], and Zimbabwe [59]. Animal fascioliasis on the other hand has been extensively reported in almost all countries in the African equatorial belt [19] and east, central, west, and southern Africa [4, 49, 50, 60, 61]. A favourable climatic and environmental picture further presents for easy human transmission (Figure 1).

On the other hand, it is strange that no published studies or reports on the disease in humans have emerged from these potentially endemic regions.

### 3.5. Treatment

Triclabendazole (TCBZ) is the drug of choice in the treatment of fascioliasis. However, in addition to the changing pattern of disease, reports of resistance to TCBZ have appeared in the literature [62–64], although they may not all represent genuine cases of resistance. Nevertheless, any reports of resistance are a concern, because TCBZ is the only drug that has shown high efficacy against the migratory and juvenile stages of infection to date [1, 3]. Resistance to the drug could potentially set back any recent gains made in the efforts to combat and manage human and animal fascioliasis.

### 3.6. Diagnosis

The diagnosis of fascioliasis has changed considerably in the 3 decades since it became a disease of primary human importance. Detection of eggs in patients' stool samples is still considered the most conclusive diagnosis at the clinical level [2, 9, 11]. New, improved coprologic antigen tests [65], serological and ELISA tests [52, 66], radiological and imaging diagnostics [30, 67, 68], and highly specific molecular techniques [26, 69] have been developed and are the current hallmark of sound and scientific studies in the field [1, 70]. Detection of resistant strains, differentiation of the acute stages from chronic stages of infection, and identifying reinfections after treatment still remain a challenge at the clinical level.

Geospatial distributions of snail vectors and parasite transmission [59, 71, 72] and of zoonotic epidemiology [3, 27] are recent areas of development in anticipating and planning for epidemics of human fascioliasis. Model drugs and interventions [62, 73] are also areas of novel research into the effective management of human and animal fascioliasis.

## 4. Issues of Immediate Concern about Fascioliasis in the Least Developed Countries

Although fascioliasis has been established as a disease of human importance, it is still considered primarily an animal disease, particularly of sheep and cattle [74]. Lack of awareness presents a major obstacle in the effective management of human fascioliasis [1, 2]. Unfortunately in many of the least developed countries burdened by poverty and infectious diseases, human fascioliasis is not a recognized and reportable disease. Awareness and sensitization are key first steps to any planned intervention strategies.

Fortunately to date there have been no reported deaths directly associated with human fascioliasis infection. This inevitably attributes a low emergency health priority to the disease, making it one of the most neglected tropical diseases [75]. The disease has also been reported to have a much higher prevalence among the female children of school going age. The associated morbidity and Disability Adjusted Life Years (DALY) impact of the disease are thus of more significant concern [16, 36]. The consequent morbidities on chronic disease significantly contribute to poor quality of life, expectancy, and productive output since the adult worm can live for over 10 years in a suitable host [4, 19].

There have been only scattered reports of outbreaks of fascioliasis in irrigated agricultural areas free from domestic animals [18, 19]. The highest proportions of reported cases are zoonotic in nature, and so any intervention strategies should ideally address the problem at the veterinary level as well. Human fascioliasis is hardly reported in those countries where animal fascioliasis is highly prevalent [4, 76, 77]. Vigilant surveillance and screening programs should be implemented in such areas, with an emphasis on interdisciplinary involvement across various professions [5, 54].

The global awareness and one health approach to zoonoses are by far the most comprehensive approach to fascioliasis [45]. Sustained efforts are still required including control measures for trade and travel to curb the spread of infected animals from one country to another. Improved water and food hygiene programs are further important components of control programs [44, 74]. Stakeholder involvement and the political will to back such strategies are crucial to the effective uptake of these interventions.

Definitive reports of drug resistance to triclabendazole [78] should be rigorously investigated and alternative treatment options sought. Vaccine development should also be an area of future research. Mixed infections with other trematode or intestinal parasites [17, 79–81] confound early detection of fascioliasis. Delayed and missed diagnoses especially among the young key population magnify the DALY impact of the disease [82, 83].

## 5. Future Challenges

The 21st century has seen a dawning in the knowledge on human fascioliasis evidenced by the number of publications on the subject over the last 10 years. The complete implementation of this new knowledge and its translation into tangible results remain a challenge in the least developed countries (LDCs). Populations in LDCs are the highest at risk of disease because of the following reasons: (i) poor access to this new body of knowledge, (ii) limited resources to put it into practical use, or (iii) having these limited resources dedicated to “more threatening” problems than fascioliasis [1, 2, 45]. The poverty-disease cycle is indeed a vicious, autocatalytic cascade.

A typical complicating infection control scenario of zoonotic infections including fascioliasis in sub-Saharan Africa includes: (a) global warming and civil unrest, (b) close proximity to domestic animals, (c) rural-urban migration with poor personal, water, and food hygiene, and (d) lax biosafety and surveillance systems. Therefore, control programs of human fascioliasis should have an integrated approach whereby all factors that contribute to the presence of the disease are considered [44, 75].

Cutting edge advances in diagnostic, surveillance, and management techniques of fascioliasis have been made. Yet the developing countries and particularly the lowest income communities are not able to access these advances because of poverty. Heavily burdened by diseases, civil unrest, and competition for scarce resources, it is not surprising that there are hardly any reports on human fascioliasis from these regions [2, 4]. Control programs should first consider rigorous awareness campaigns and sensitization on both the magnitude and impact of human fascioliasis in humans and animals [44].

The current “One Health Integrated Global Approach to Disease” presents by far the most comprehensive and participatory solution, not only to human fascioliasis but also to the bulk of zoonotic diseases at large [44, 85]. A classic example of the problems it tackles can best be elaborated in the recent drive to “Go Green,” as a healthy approach to the modern artificial lifestyle. Compartmentalized to specific sections like nutrition and preventive medicine, agriculture, and industry, this has seen an unprecedented increase in the consumption of fresh, raw/green fruit and vegetables [86, 87]. This is however poorly backed by water safety, fertilizer-pesticide use control, and waste management. The consumption of poorly monitored, produced, and stored fresh green vegetables has contributed to the increased spread of plant/food-borne trematodiases including fascioliasis, among many other health problems [48, 83].

Controlled clinical trials to investigate reported cases of triclabendazole and bithionol resistance of* Fasciola* are areas of immediate research interest. Further development of chemotherapeutic options like the Myrrh-derived Mirazid and nitazoxanide [19, 73, 88], as well as other novel interventions aimed at the intermediate snail hosts [71, 89], may provide much needed alternative chemotherapy. Control strategies aimed at the animal reservoirs and active surveillance for disease hotspots allow early intervention while improved food and water safety combined with possible vaccine development is vital to prevention strategies of human fascioliasis. In order to succeed, all this needs to be backed by rigorous awareness, sensitization campaigns and political will to maximize uptake [75, 90].

Human fascioliasis is perceived as a low significance “Neglected Tropical Disease of Poverty” [16, 42, 45, 87]. As interventions and solutions to the disease are developed in the more developed countries/communities, support structures, basic amenities, and simple interdisciplinary collaborations degenerate equally fast in the lowest income communities at particularly high risk of infection [74, 75]. A case in point is observed in the abundance of veterinary reports on animal fascioliasis out of sub-Saharan African countries, countered by an almost total disregard for the human zoonoses among the medical and public health community [49, 50, 53]. It is surprising that human fascioliasis is still not a reportable disease in many of these countries.

However, because of easy and fast global travel currently prevailing, open markets and free trade, cultural tourism, and massive cultural and national integration, the problems of the developing countries may spill to more developed countries [39, 45, 46]. The dawn of unpredictable climate changes and their effect on ecobiology, civil unrest, and the simple natural laws of evolution are factors that have altered the patterns of spread of zoonoses [47, 48]. For example, the recent global threat from the West African Ebola outbreak is a fresh reminder of the far reaching ramifications of unexpected disease outbreaks on the continent [75, 91]. Human fascioliasis is still nonfatal and results from interventions used in most hyperendemic regions prove that it can be effectively controlled, if not eradicated. As an NTD, this should be a tangible target [74, 87].

## 6. Conclusion

The fact that human fascioliasis reporting in the least developed nations is lacking presents a particularly difficult challenge. These countries are already heavily burdened by different diseases and lack access to adequate resources. Therefore, despite major progress in the diagnosis and control of human fascioliasis in the more developed countries, the disease continues to be a significant public health problem [84, 92].

The breakdown of interdisciplinary collaborations, coupled with political and civil unrest, further perpetuates the prevalence of many diseases including fascioliasis in the developing countries. We propose that control measures of fascioliasis should include (i) general and clinical awareness, (ii) integrated and multidisciplinary collaborations, (iii) sustained interventions backed by political will, and (iv) vaccine and drug development.
